# Comparative Effectiveness of Closed Reduction With Percutaneous Pinning and Open Reduction With Internal Fixation in the Operative Management of Pediatric Type III Supracondylar Fractures

**DOI:** 10.7759/cureus.22707

**Published:** 2022-02-28

**Authors:** Mohammad A Abousaleh, Anas A Zeidan, Iftikhar Mukhtar, Ahmed S Keshta, Taibah H Aladraj, Omaima A Shaaban, Mohamed S Keshta, Rashad Alqasim

**Affiliations:** 1 Orthopaedic Surgery, Royal College of Surgeons in Ireland - Bahrain, Busaiteen, BHR; 2 Pediatric Orthopaedics, Royal College of Surgeons in Ireland - Bahrain, Busaiteen, BHR; 3 Pediatric Orthopaedics, Bahrain Defense Force Hospital, Riffa, BHR; 4 Pediatrics, Royal College of Surgeons in Ireland - Bahrain, Busaiteen, BHR; 5 Orthopaedics, Royal College of Surgeons in Ireland - Bahrain, Busaiteen, BHR; 6 Anaesthesia, Bahrain Defense Force Hospital, Riffa, BHR

**Keywords:** closed reduction, open reduction, pediatric type iii supracondylar fractures, supracondylar humeral fractures, type iii supracondylar fractures, closed reduction with percutaneous pinning, open reduction internal fixation

## Abstract

Background

Supracondylar fracture with total displacement is classified as Gartland type 3. The operative management for this type of fracture can be closed reduction with percutaneous pinning (CRPP) or open reduction with internal fixation (ORIF). This study aims to determine whether CRPP or ORIF led to smaller changes in Baumann’s angle, the carrying angle, loss of motion, and complication when treating pediatric supracondylar fractures.

Methodology

In a retrospective cohort design, pediatric patients presenting with supracondylar fractures at a tertiary care hospital in Bahrain between March and October of 2021 were enrolled. The collected data included age, gender, nationality, mechanism of injury, neurovascular status, type of surgery performed, follow-up period, range of motion, complications, Baumann’s angle, carrying angle, and loss of motion. The changes in Baumann’s angle, carrying angle, and reduction sufficiency were compared to the literature using Flynn’s criteria for supracondylar fractures.

Results

This study included the records of 60 patients with supracondylar fractures. In total, 28 patients underwent CRPP (group A), whereas 32 underwent ORIF (group B). A statistically significant difference (p = 0.037) between group A and group B was noted when combining the loss of carrying angle scores and the loss of motion scores to form the final Flynn score. In group A, 26 (92.8%) cases had satisfactory results; 75% of these cases were excellent or good. According to Flynn’s criteria, all patients in group B were satisfactory; 93.75% of these cases were excellent or good. The loss of motion was significantly different between the two groups (p = 0.038). The mean loss of carrying angle was significantly different between the two groups, with 5.51 ± 3.03 degrees for group A and 4.23 ± 1.85 degrees for group B (p = 0.023). The study had only two cases with unsatisfactory ratings belonging to group A.

Conclusions

In pediatric patients presenting with type 3 supracondylar fractures, when compared to CRPP, ORIF was associated with less loss of motion, less loss of carrying angle, higher overall satisfactory results according to Flynn’s criteria, and fewer complications.

## Introduction

Supracondylar fractures occur at the distal end of the humerus bone in the arm and are the second most common fracture in children, accounting for 3-18% of all fractures [1,2]. In the humerus, the weakest point is where the lateral column merges with the olecranon fossa and, therefore, carries the highest fracture risk. Because elbow injuries are prevalent in the pediatric population due to increased falls, supracondylar fractures are most common in children aged five to seven years [3]. Several studies conducted among various age groups have concluded that children under the age of seven represent the highest incidence group. Most studies divide children’s age groups into two categories, zero to three years old and three to seven years old. According to the literature, children under the age of three years account for 28.9% of supracondylar fractures. Overall, 31.2% and 53.6% of supracondylar fractures occur in children aged four to seven and three to six years, respectively [1,3,4].

Based on severity, bone displacement, and neurovascular damage, pediatric supracondylar fractures are graded into three main categories using the Gartland classification [5]. Undisplaced fractures are classified as Gartland type 1 and indicate a minor severity level. Displaced fractures that remain intact with a hinge posteriorly are type 2 Gartland fractures [5]. Supracondylar fractures with total bone displacement are type 3 Gartland fractures [5].

Almost 49% of supracondylar fractures have accompanying neurovascular or vascular injuries. Therefore, suspected supracondylar fractures strongly warrant a neurological and vascular examination of the affected limb [3]. As high as 38% of Gartland type III fractures are associated with brachial artery injury [6], whereas radial nerve injury occurs in up to 28%. All Gartland type III fractures are associated with posteromedial displacement [6].

The management of pediatric supracondylar fractures depends on the Gartland classification and the presence of neurovascular dysfunction [7]. For type 3 fractures, operative management can be in the form of closed reduction with percutaneous pinning (CRPP) or open reduction with internal fixation (ORIF) [8]. There is sufficient evidence supporting CRPP to be as beneficial as ORIF with fewer associated complications in cases where ORIF is not indicated. ORIF is indicated in cases when CRPP fails, open fractures, neurovascular compromise cases, and late presentation cases [8,9]. The advantages of CRPP include less trauma, shorter hospital stay, reduced postoperative stiffness, and faster recovery [8,10]. Chang et al. reported ORIF to be associated with a smaller change in Baumann’s angle [11]. A study done by Musa et al. found that 97% of the children with type III Gartland fracture who underwent CRPP had satisfactory results, with only one case reporting a poor result [12]. Currently, there is a shortage of evidence to summarize and compare CRPP and ORIF in terms of postoperative outcomes in the pediatric population, especially in the local population in Bahrain. This warrants an initial investigation to understand the comparative effectiveness of the two procedures in the local population. This study aims to compare the postoperative outcomes of CRPP and ORIF, specifically, the range of motion of the joint, change in carrying angle, and occurrence of overall complications.

## Materials and methods

Location

This retrospective cohort study was conducted at Bahrain Defense Force (BDF) Hospital over seven months from March 2021 to October 2021.

Sample size and selection

Patients below the age of 18 who attended BDF Hospital with a type III supracondylar Gartland fracture between 2015 and 2021 were included in the study. Data were obtained from the hospital’s electronic records. A total of 60 patients under 18 with Gartland type III supracondylar fractures were included.

Cohorts

All patients were seen in the emergency room and stabilized. All patients were treated surgically, either by CRPP or ORIF. Patients who underwent CRPP are referred to as group A. Patients who underwent ORIF are referred to as group B.

Data collection

The medical records of each patient were evaluated to obtain data on age, gender, nationality, mechanism of injury, neurovascular status, type of surgery performed, follow-up period, range of motion, and complications. Radiographic images were analyzed for data regarding the bone’s structural deficit and postoperative improvement. The radiological outcomes measured include Baumann’s angle, carrying angle, and reduction sufficiency. These outcomes were assessed and compared to the literature using Flynn’s criteria (Table 1) [13]. Baumann’s angle was identified and measured as the angle formed by the humeral axis and a straight line through the epiphyseal plate of the capitulum [14]. Baumann’s angle was compared at immediate post-reduction and post-union [15]. The carrying angle was identified and measured as formed between the axis of a radially deviated forearm and the axis of the humerus [16]. The change in the carrying angle and the loss of motion was determined by comparing the affected arm with the unaffected arm [17]. The final Flynn’s score was based on the greater clinical loss if the patient had different carrying angles and range of motion scores. For example, a good score in the loss of motion rating and a fair score in the change of carrying angle resulted in a fair final Flynn’s score [15]. This retrospective cohort study was approved by the Research Ethics Committee of the Royal Medical Services - Bahrain Defense Force (RMS-BDF) on Match 14, 2021.

**Table 1:** Flynn’s criteria.

Results	Rating	Loss of carrying angle	Loss of motion
Satisfactory	Excellent	0°-5°	0°-5°
	Good	5°-10°	5°-10°
	Fair	11°-15°	11°-15°
Unsatisfactory	Poor	>15°	>15°

Statistical analysis

Continuous variables have been represented as mean and standard deviation. Discrete variables have been expressed as frequencies and percentages. For continuous variables, Student’s t-test or Mann-Whitney U test was used, depending on the results from the Shapiro-Wilk test of normality. The chi-square test and Fisher’s exact test were used for categorical variables. P-values less than 0.05 were considered statistically significant. All analyses were performed using SPSS version 25 (IBM Corp., Armonk, NY, USA).

## Results

In total, 60 cases of Gartland 3 supracondylar fracture were evaluated. Of these, 28 (47%) cases were operated by CRPP (group A) and 32 (53%) cases by ORIF (group B). Table 2 shows the participants’ demographic characteristics. The overall age range was 1-16 years (6 ± 3.37), with ages of participants in group A ranging 1-9 years (5.21 ± 2.17) and those in group B ranging 2-16 years (6.69 ± 4.08, p = 0.403). The male-to-female ratio was 1:1 in group A and 1.9:1 in group B (p = 0.221). Overall, 38 (63%) cases sustained the fracture to their left upper limb and 22 (37%) cases to their right upper limb. The mechanism of injury was fall in 52 (87%) cases, seven (11%) cases were injured due to trauma while playing, and one (2%) case sustained the fracture in a road traffic accident. The overall average time between the injury and the surgical operation was 1.77 ± 2.66 days; 1.33 ± 1.54 days for group A and 2.15 ± 3.31 days for group B (p = 0.842). The average hospital stay was 1.17 ± 0.71 days, with no significant difference between the two groups. The average metal removal duration was 2.76 ± 4.67 months, and the average cast removal duration was 1.92 ± 1.54 months, with no significant difference between the two groups. The overall average follow-up time was 5.92 ± 8.41 months; 3.64 ± 2.61 months for group A and 8.13 ± 11.16 months for group B (p = 0.276). Three (5%) cases developed complications, two in group A and one in group B. Two out of the three complications were ulnar nerve injury (one case in group A and one case in group B), and the third complication was a hypertrophic scar in a group A case. All neural injuries resolved within 12 weeks. None of the cases developed a vascular injury or an infection. Physiotherapy was undertaken in seven (25%) cases in group A and 18 (56%) cases in group B.

**Table 2:** Demographic data comparing groups A and B. M = mean; SD = standard deviation; CRPP = closed reduction with percutaneous pinning; ORIF = open reduction with internal fixation; ^‡^ = Mann-Whitney U test; ^a ^= chi-square test; ^b^ = Fisher’s exact test

Parameter	Overall sample (N = 60)	CRPP (group A) (n = 28)	ORIF (group B) (n = 32)	P-value (ORIF vs. CRPP)
Age (mean ± SD) (years)	6 ± 3.37	5.21 ± 2.17	6.69 ± 4.08	0.403^‡^
Gender (male: female)	1.4:1	1:1	1.9:1	0.221^a^
Mechanism of injury: Fall (cases)	52	26	26	>0.05^b^
Mechanism of injury: Trauma while playing (cases)	7	2	5	
Mechanism of injury: RTA (cases)	1	0	1	
Side of injury (right: left)	22:38	11:17	11:21	0.694^a^
Time between injury and surgery (mean ± SD) (days)	1.77 ± 2.66	1.33 ± 1.54	2.15 ± 3.31	0.842^‡^
Hospital stay time (mean ± SD) (days)	1.17 ± 0.71	1.18 ± 0.94	1.16 ± 0.45	0.240^‡^
Metal removal time (mean ± SD) (months)	2.76 ± 4.67	1.60 ± 1.61	3.78 ± 6.07	0.070^‡^
Cast removal time (mean ± SD) (months)	1.92 ± 1.54	1.95 ± 1.68	1.90 ± 1.44	0.892^‡^
Follow-up period (mean ± SD) (months)	5.92 ± 8.41	3.64 ± 2.61	8.13 ± 11.16	0.276^‡^
Overall complications (cases)	3	2	1	0.594^b^
Ulnar never injuries (cases)	2	1	1	>0.05^b^
Hypertrophic scar (cases)	1	1	0	0.467^b^
Vascular injury (cases)	0	0	0	-
Infections (cases)	0	0	0	-
Physiotherapy done	25	7	18	0.129^a^
Physiotherapy not done	35	21	14	

The clinical and radiological outcomes of groups A and B are presented in Table 3 with their p-values. The clinical and radiological results were categorized according to Flynn’s criteria (Table 1). The mean Baumann’s angle measured post-union was 68.02 ± 9.83 degrees for group A and 70.75 ± 6.90 degrees for group B (p = 0.341). Baumann’s angle at any point in time and the change in Baumann’s angle were not significantly different between the two groups. The average carrying angle measured post-union was 8.51 ± 3.13 degrees for group A and 8.38 ± 2.86 degrees for group B (p = 0.620). The mean loss of carrying angle was significantly different between the two groups, with 5.51 ± 3.03 degrees for group A and 4.23 ± 1.85 degrees for group B (p = 0.023). Overall, 59 (98.3%) cases had satisfactory results, with 93.3% graded as excellent or good according to Flynn’s criteria for the carrying angle. In group A, 27 (96.4%) cases had satisfactory results according to Flynn’s criteria for loss of motion (i.e., loss of motion of fewer than 15 degrees), and only one case had unsatisfactory results (i.e., loss of motion of >15 degrees). In group B, all cases had satisfactory results according to Flynn’s criteria for loss of motion (i.e., loss of motion of fewer than 15 degrees), with 87.5% of these cases graded as excellent (i.e., loss of motion less than 5 degrees). The loss of motion was significantly different between the two groups (p = 0.038). A statistically significant difference (p = 0.037) between groups A and B was noted when the loss of carrying angle scores and the loss of motion scores were combined to form the final Flynn score. In group A, 26 (92.8%) cases had satisfactory results; 75% of these cases were excellent or good. According to Flynn’s criteria, all patients in group B were satisfactory; 93.75% of these cases were excellent or good. The study had only two cases with an unsatisfactory rating, both of which belonged to group A.

**Table 3:** Comparison of the clinical and radiological outcomes between groups A and B. M = mean; SD = standard deviation; CRPP = closed reduction with percutaneous pinning; ORIF = open reduction with internal fixation; * = significant at the 0.05 level; ^‡^ = Mann-Whitney U test; ^†^ Student’s t-test; ^a^ = chi-square test; ^b^ = Fisher’s exact test

Parameter			Overall sample (N = 60)	CRPP (Group A) (n = 28)	ORIF (Group B) (n = 32)	P-value
Change in Baumann’s angle (mean ± SD) (degrees)			6.98 ± 5.46	8.21 ± 6.33	5.90 ± 4.39	0.343^‡^
Baumann’s angle post-surgery (mean ± SD) (degrees)			69.48 ± 8.55	68.02 ± 9.83	70.75 ± 7.17	0.231^†^
Baumann’s angle post-union (mean ± SD) (degrees)			73.53 ± 7.38	72.56 ± 7.90	74.39 ± 6.90	0.341^†^
Baumann’s angle change (Flynn’s criteria): Number of cases	Satisfactory	Excellent (0°–5°)	32	12	20	0.223^b^
		Good (5°–10°)	12	5	7	
		Fair (11°–15°)	11	8	3	
	Unsatisfactory	Poor (>15°)	5	3	2	
Carrying angle post-union (mean ± SD) (degrees)			8.44 ± 2.97	8.51 ± 3.13	8.38 ± 2.86	0.620^‡^
Loss of carrying angle (mean ± SD) (degrees)			4.83 ± 2.53	5.51 ± 3.03	4.23 ± 1.85	0.023^‡^*
Loss of carrying angle (Flynn’s score): number of cases	Satisfactory	Excellent (0°–5°)	49	21	28	0.608^b^
		Good (5°–10°)	7	4	3	
		Fair (11°–15°)	3	2	1	
	Unsatisfactory	Poor (>15°)	1	1	0	
loss of motion (Flynn’s score): number of cases	Satisfactory	Excellent (0°–5°)	44	18	26	0.038^b^*
		Good (5°–10°)	10	4	6	
		Fair (11°–15°)	5	5	0	
	Unsatisfactory	Poor (>15°)	1	1	0	
Final Flynn’s score: number of cases	Excellent		40	16	24	0.037^b^*
	Good		11	5	6	
	Fair		7	5	2	
	Poor		2	2	0	
Flynn’s satisfactory score: number of cases	Satisfactory		58	26	32	0.214^b^
	Unsatisfactory		2	2	0	

## Discussion

Supracondylar fractures represent a large proportion of elbow fractures in the pediatric population. Immediate reduction is indicated because inadequate reduction can result in cubitus varus [18]. The displaced fragment can damage nearby structures leading to artery transection, thrombosis, or reduced arterial flow causing Volkmann’s ischemic contracture [19]. The literature has clear guidelines regarding Gartland types 1 and 3 management, but type 2 is surgeon and injury-dependent. Type 3 Gartland fractures are managed surgically in all cases. However, the ambiguity lies in the type of surgery. In theory, CRPP is quicker and associated with fewer complications, whereas ORIF gives the additional options of resecting foreign bodies and dissecting hematomas [18].

This study compared functional outcomes of CRPP and ORIF in the surgical management of pediatric supracondylar fractures. In addition, the study aimed to identify which technique yields more significant results for better treatment with fewer complications. Like all surgical specialties, orthopedic surgery seeks to produce the best results with the fewest complications and effects on the patient. More extensive surgeries require higher doses of anesthesia, longer hospitalization time, and have higher complication incidence. This research analyzed whether CRPP leads to similar reduction results without the complications associated with ORIF. In our study, 28 (47%) cases were operated by CRPP (group A) and 32 (53%) cases by ORIF (group B). In previous literature, the percentage of cases that needed ORIF varied from 1.3% to 46% [18-20].

The most significant finding was that ORIF led to significantly better results than CRPP when using the final Flynn’s score (p = 0.037). The score includes the loss of carrying angle and motion scores. A loss of motion of greater than 15 degrees is unsatisfactory, while a loss of fewer than 15 degrees is satisfactory. Regarding our study’s loss of motion criteria, 96.4% of the patients who underwent CRPP had satisfactory results, while the rest were deemed unsatisfactory. In comparison, 100% of patients who underwent ORIF had satisfactory results regarding the loss of motion. The difference between the two groups regarding loss of motion was significant (p = 0.038), favoring group B (ORIF). The loss of carrying angle was less in group B (4.23 degrees) than 5.51 for group A, with statistical significance (p = 0.023). Overall, 57% in group A were labeled as excellent in the final Flynn’s score compared to 75% of those in group B, deeming the latter more superior (p = 0.037). Two (7%) cases of group A scored a poor final Flynn’s score compared to none in group B. The two cases that achieved poor final Flynn’s scores in group A are the only ones in the entire study with poor final Flynn’s scores. One of these cases had a loss of carrying angle greater than 15 degrees (poor) while having a loss of motion between 10 and 15 degrees (fair), deeming the final score as poor. The other case with poor final Flynn’s score had a poor loss of motion (>15 degrees) and fair loss of carrying angle (10-15 degrees), deeming the final score as poor. According to previous literature, the range of CRPP (group A) that had excellent results was 57% to 81%, significantly higher than those in our study who received CRPP [13,21,22]. They also report a poor outcome in CRPP ranging from 2% to 14%, which correlates well with our results. As shown in Table 4, patients in our study who underwent CRPP had less favorable outcomes than those who underwent the same procedure in the study done by Musa et al. [12]. A study was done in 2000 by Mulhall et al. to study the outcomes of completely displaced supracondylar fractures in children. In our research, 16 patients were included with a mean age of 5.9 years (compared to their study, which included 32 patients with a mean age of 6.69 years). All patients in the mentioned study were treated with ORIF [23]. The cited research used Flynn’s criteria to assess its outcomes, making it comparable to our study. A comparison between the two studies is shown in Table 5. According to the final Flynn’s score, both studies had the highest percentage of outcomes as “excellent” (75% and 81.25% in our research and Mulhall et al., respectively). In addition, both studies had zero cases classified as poor outcomes according to Flynn’s criteria [23].

**Table 4:** Comparison of this paper’s CRPP results with the literature. CRPP = closed reduction with percutaneous pinning

			Our paper	Musa et al. [12]
Baumann’s angle change (Flynn’s criteria): number of cases, CRPP	Satisfactory	Excellent (0°–5°)	12/28 (43%)	27/30 (90%)
		Good (5°–10°)	5/28 (18%)	1/30 (3%)
		Fair (11°–15°)	8/28 (28%)	2/30 ( 7 %)
	Unsatisfactory	Poor (>15°)	3/28 (11%)	0/30 (0%)

**Table 5:** Comparison of this paper’s ORIF results to a similar study. ORIF = open reduction and internal fixation

			Our paper	Mulhall et al. [23]
Final Flynn’s score: number of cases-number of cases, ORIF	Satisfactory	Excellent	24/32 (75%)	13/16 (81.25%)
		Good	6/32 (18.75%)	2/16 (12.5%)
		Fair	2/32 (6.25%)	1/16 (6.25%)
	Unsatisfactory	Poor	0/32 (0%)	0/16 (0%)

The average age of the patients was 5.21 years in group A and 6.99 years in group B. Similar papers in the literature have reported slightly higher average ages, with group A having an average age ranging from 5.4 to 7.06 years and group B between 6.1 to 7.2 years [12,16,19]. In this study, the male-to-female ratio was 1:1 for group A and 1.9:1 for group B. A similar study comparing CRPP and ORIF in pediatrics had a much lower male-to-female ratio; group A 4:11 and group B 5:9 [18]. The overall male-to-female ratio in our study was 1.4:1, which is consistent with a study done by Cheng et al. in 2001 in which boys were found to be more prone to supracondylar fractures after the age of four [24]; however, this contrasts with a North American study which found that girls are more prone to such fractures [25]. Our study had three overall complications (5%). Two complications were ulnar nerve injuries (one case in group A and one case in group B). The two patients had palsy in the fourth and fifth fingers with reduced sensation. Nerve conduction studies were performed. All neural injuries resolved within 12 weeks. A population-based study done in 2014 found that patients who underwent ORIF were more likely to undergo early nerve exploration and re-operation in the long term compared to CRPP [26]. Our study’s third patient with a complication belonged to group A and had tender and prominent K-wires with a hypertrophic scar. We did not have any pin site infection compared to Cramer et al., who had 6% in group A and 7% in group B. In our study, the overall time between injury and surgery was 1.77 ± 2.66 days (1.33 ± 1.54 days and 2.15 ± 3.31 days for groups A and B, respectively) (Table 2). Many previous studies have expressed that the emergent treatment of these fractures is necessary to avoid infectious, neurological, or vascular complications [21,27,28].

This study has several strengths, the most prominent one being that this work is the first of its kind to be conducted in Bahrain. The variables compared allowed for a comprehensive study of the complications and outcomes.

The sample size and the nature of the study (retrospective cohort) are two limitations of the study. Larger sample size would amplify the results and clearly demarcate the superiority between the two approaches. Retrospective cohorts are a limitation in themselves due to the possible loss of data in the medical records.

## Conclusions

Although both techniques displayed impressive results in terms of reduction capabilities and complications, ORIF was superior. ORIF was associated with less loss of range of motion, less loss of carrying angle, and significantly higher overall satisfactory results according to Flynn’s criteria. CRPP had cases of poor final Flynn’s scores, while ORIF did not. Future research should be undertaken on larger populations, amplifying the rare findings. This, in turn, will lead to more understanding of the differences between the two management approaches. A future study with constant variables such as the extent of injury, age, gender, and etiology will provide more concrete evidence of which management technique yields more satisfactory results.
